# Green Synthesized Zinc Oxide Nanoparticles Using *Moringa olifera* Ethanolic Extract Lessens Acrylamide-Induced Testicular Damage, Apoptosis, and Steroidogenesis-Related Gene Dysregulation in Adult Rats

**DOI:** 10.3390/antiox12020361

**Published:** 2023-02-02

**Authors:** Gomaa Mostafa-Hedeab, Amany Behairy, Yasmina M. Abd-Elhakim, Amany Abdel-Rahman Mohamed, Ahmed E. Noreldin, Naief Dahran, Rasha A. Gaber, Leena S. Alqahtani, Walaa M. Essawi, Areej A. Eskandrani, Eman S. El-Shetry

**Affiliations:** 1 Pharmacology Department & Health Research Unit, Medical College, Jouf University, Sakaka 72446, Saudi Arabia; 2 Department of Physiology, Faculty of Veterinary Medicine, Zagazig University, Zagazig 44511, Egypt; 3 Department of Forensic Medicine and Toxicology, Faculty of Veterinary Medicine, Zagazig University, Zagazig 44519, Egypt; 4 Department of Histology and Cytology, Faculty of Veterinary Medicine, Damanhour University, Damanhour 22511, Egypt; 5 Department of Anatomy, Faculty of Medicine, University of Jeddah, Jeddah 23218, Saudi Arabia; 6 Medical Biochemistry Department, Faculty of Medicine, Tanta University, Tanta 31527, Egypt; 7 Department of Biochemistry, College of Science, University of Jeddah, Jeddah 80203, Saudi Arabia; 8 Department of Theriogenology, Faculty of Veterinary Medicine, Aswan University, Aswan 81528, Egypt; 9 Chemistry Department, College of Science, Taibah University, Medina 30002, Saudi Arabia; 10 Department of Human Anatomy and Embryology, Faculty of Medicine, Zagazig University, Zagazig 44511, Egypt

**Keywords:** acrylamide, green synthesis, zinc oxide nanoparticles, male fertility, oxidative stress, apoptosis, steroidogenesis genes, rats

## Abstract

This study assessed the possible protective role of green synthesized zinc oxide nanoparticles using *Moringa olifera* leaf extract (MO-ZNPs) in acrylamide (ACR)-induced reproductive dysfunctions in male rats. ACR (20 mg/kg b.wt/day) and/or MO-ZNPs (10 mg/kg b.wt/day) were given orally by gastric gavage for 60 days. Then, sperm parameters; testicular enzymes; oxidative stress markers; reproductive hormones including testosterone, luteinizing hormone (LH)-estradiol, and follicle-stimulating hormone (FSH) concentration; testis histology; steroidogenesis-related gene expression; and apoptotic markers were examined. The findings revealed that MO-ZNPs significantly ameliorated the ACR-induced decline in the gonadosomatic index and altered the pituitary–gonadal axis, reflected by decreased serum testosterone and FSH with increased estradiol and LH, and sperm analysis disruption. Furthermore, a notable restoration of the tissue content of antioxidants (catalase and reduced glutathione) but depletion of malondialdehyde was evident in MO-ZNPs+ACR-treated rats compared to ACR-exposed ones. In addition, MO-ZNPs oral dosing markedly rescued the histopathological changes and apoptotic caspase-3 reactions in the testis resulting from ACR exposure. Furthermore, in MO-ZNPs+ACR-treated rats, ACR-induced downregulation of testicular steroidogenesis genes and proliferating cell nuclear antigen (PCNA) immune-expression were reversed. Conclusively, MO-ZNPs protected male rats from ACR-induced reproductive toxicity by suppressing oxidative injury and apoptosis while boosting steroidogenesis and sex hormones.

## 1. Introduction

Acrylamide (ACR) is a crystalline, colorless, and odorless monomer used mainly to produce polyacrylamide with wide industrial applications, including dyes, plastics, paper products, and drinking water and wastewater treatment [1,2]. It is also found in various consumer products such as food packaging, caulking, and adhesives [3]. Additionally, ACR is spontaneously formed during the thermal treatment of foods containing the amino acid asparagine, such as biscuits, French fries, coffee, chips, bread, breakfast cereals, and chocolate [4,5]. It is also a component of tobacco smoke [6].

Because of its hydrophilic nature, ACR is rapidly absorbed through the gastrointestinal tract and transported to different body organs [7]. In the body, ACR is metabolized by either conjugation with glutathione (GSH), forming metabolites excreted by the urine [8], or oxidation with cytochrome P450 2E1, forming the genotoxic metabolite glycidamide [9]. Furthermore, ACR could react with proteins containing thiol (SH) and amine (NH_2_) groups of cell macromolecules, resulting in reactive oxygen species (ROS) overproduction, oxidative stress, and further DNA damage [10]. Therefore, long-term ACR exposure has been shown to cause testicular toxicity [11,12], genotoxicity [13,14], neurotoxicity [15,16], hepatotoxicity, and nephrotoxicity [17].

Various negative impacts on male reproduction functions have been associated with ACR exposure. For instance, Wang et al. [18] reported that male rats were orally given ACR at doses of 10 mg/kg/day for eight weeks and showed decreased sperm reserves in the epididymis and testicular histopathological lesions, indicating partial germ cell depletion. Furthermore, testicular cytotoxicity, including unusual giant cell formation, seminiferous tubule atrophy, and apoptotic reactions, has been linked with ACR exposure [19]. Moreover, ACR has been reported to disrupt the Leydig cells’ steroidogenic pathway and subsequently decrease testosterone levels [20]. 

Currently, nanoparticles (NPs) are used in numerous fields, including disease diagnostics, imaging, gene sensing, drug delivery, electronics, agriculture, food industries, and electro-technology [21,22]. Because of their larger surface area and ability to easily penetrate cell membranes and barriers such as the placental barrier, blood–brain barrier, and intestinal barrier, NPs interact more efficiently with organic and inorganic substances in the animal body [23,24]. As a result, many NP preparations have exhibited great efficiency in combating several health disorders [25,26]. Zinc oxide nanoparticles (ZNPs) have received attention among metal nanosystems due to their high photo-oxidizing and photocatalytic activity [27]. ZNPs have been demonstrated to have anti-inflammatory, anticancer, antibacterial, antidiabetic, larvicidal, antifungal, and wound-healing activities [28,29,30,31,32]. Physical, chemical, and hydrothermal methods are usually used to produce ZNPs. However, these techniques use non-biocompatible, toxic, and expensive chemicals and have a negative environmental impact [33]. The earlier drawbacks highlighted green synthesis’s importance in producing low-toxic, safe, and environmentally friendly NPs [34]. Plant extracts have shown great success as a reducing agent that transforms inorganic Zn into ZNPs [31,35]. 

*Moringa oleifera* L (MO) is an important medicinal plant that grows throughout the world’s tropics and subtropics [36]. The leaves of MO have ample beneficial phytochemicals, including tannins, alkaloids, terpenoids, isothiocyanates, flavonoids, sterols, glucosinolates, anthraquinones, saponins, and glycoside compounds [37]. This gives repro-protective [38], neuroprotective [39], immunomodulatory [40], nephroprotective [41], hepatoprotective [42], antiobesity [43], anticancer [44], and anti-inflammatory [45] activities of MO leaves. Previous research has shown that ZNPs synthesized using MO leaves (MO-ZNPs) have superior antioxidant, anti-inflammatory, and antiapoptotic effects [46,47]. 

However, to our knowledge, no data exist on the possible beneficial effect of green biosynthesized MO-ZNPs on ACR-induced reproductive toxicity in the male rat paradigm. Hence, biocompatible ZnONP was synthesized using MO extract. Rats were then orally gavaged ACR and/or MO-ZNPs for 60 days to detect whether MO-ZNPs could protect against ACR-induced impairments in semen quality, male sex hormones, steroidogenesis-related gene expression, testicular oxidative status, apoptotic reactions, Zn content, and histological architecture.

## 2. Materials and Methods

### 2.1. Preparation of M. oleifera Ethanol Extracts

*M. oleifera* leaves were obtained from the Egyptian Scientific Society of Moringa, National Research Center, Egypt. The leaves were thoroughly washed with distilled water and air-dried for three weeks at room temperature, according to Okechukwu et al. [48]. The leaves were crushed to a coarse texture by an acrestor high-speed milling machine and were then macerated for 48 h in absolute ethanol before filtration. Concentrating and dehydrating the resulting ethanol extract required a rotary evaporator heated to 40–45 °C. Distilled water containing 1% carboxy methyl cellulose was used to dilute the extract before refrigeration. The extract was used in the subsequent synthesis of ZNPs as a reducing agent.

### 2.2. Green Synthesis and Characterization of MO-ZNPs

A 10 mL aliquot of the *M. oleifera* leaf extract was diluted with 80 mL of distilled water and heated to 80 °C while stirring. Extract of *M. oleifera* was mixed with 20 mL of a 1% Zn acetate (Sigma-Aldrich Co., St. Louis, MO, USA) solution in water. While constantly stirring, the *M. oleifera*-Zn solution was heated for 4 hrs. A few drops of 0.1 M NaOH were added to make the solution slightly alkaline, and stirring was maintained for an additional 2 hrs. The synthesis of ZNPs was indicated by a change in solution hue to dark greenish and turbid, with little white precipitate [49]. A little precipitate formed in the MO-ZNPs dispersion right after the reduction, suggesting that the particles were aggregating or larger. Nonetheless, the suspension was relatively stable, keeping its turbidity and dark hue for over a month.

By a high-resolution transmission electron microscope (HR-TEM, JEM-2100, JEOL, Tokyo, Japan), the MO-ZNPs’ particle shape and size were investigated. Zeta Sizer (Nano-ZS, Malvern Instruments Ltd., Zetasizer Ver, Malvern, UK) was utilized to evaluate the prepared ZNPs’ zeta potential, particle size and polydispersity index. Using FTIR spectroscopy (Nicolet Avatar FTIR 230, Richmond Scientific Ltd., Lancashire, UK), the surface functional groups of MO-ZNPs were identified in the 400–4000 cm^−1^ range. The elemental composition of the resulting NPs was evaluated via measuring their Edx using a field emission scanning electron microscope (FE-SEM) (QUANTA 250, FEI Company, Eindhoven, The Netherlands). The synthesized MO-ZNPs characteristics were previously presented in our earlier work [16].

### 2.3. Animals and Experimental Design

A total of 40 Sprague Dawley rats (male, 160 ± 0.20 g, 12 weeks of age) were obtained from the Laboratory Animal Housing Unit, Faculty of Veterinary Medicine, Zagazig University, Egypt. In a well-ventilated room, rats were placed in a stainless-steel cage with free access to food and water on a 12-h light/12-h dark cycle. The animals used in these investigations had two weeks to acclimatize to the laboratory environment before being used in the trial. Rats were arbitrarily alienated into four groups (10 rats per treatment). The control group was orally given 1 mL distilled water/rat for 60 days. The MO-ZNPs group received MO-ZNPs (10 mg/kg b.wt) orally [46]. The ACR group was orally dosed ACR (CAS No. 79-06-1, Sigma-Aldrich Co., St. Louis, MO, USA, dissolved in distilled water) at a concentration of 20 mg/kg body weight [50]. All treatments were given orally using gastric gavage. The ACR+MO-ZNPs group received the ACR and MO-ZNPs orally at the previously stated doses with a one-hour interval between doses for 60 days. Every rat was weighed once a week, and the dose volumes were adjusted based on the new weight. The rats were carefully observed throughout the experiment for respiratory patterns, pain, discomfort, injury, mucous membrane color, morbidity, and mortality.

### 2.4. Blood and Tissue Sampling

Following intraperitoneal administration of 50 mg/kg b.wt of ketamine hydrochloride and xylazine, rat weights were recorded, and the animals were euthanized. Rats’ blood was collected through retro-orbital venous plexus puncture and centrifuged in clean, sterile, nonheparinized tubes. After letting the blood clot for 15 min, it was centrifuged at 3000 rpm to separate the serum. The clear serum was kept at −20 °C until use for hormonal analysis. The rats were euthanized after blood samples were taken, and the testicles were quickly removed, cleaned, and weighed using an automated analytical balance. To determine the gonadosomatic index, the average testicle weight was divided by the body weight × 100 [51]. 

Necropsied testicular samples were separated into three groups. The first group was frozen at −80 °C for gene expression analysis. The second one was homogenized in chilled potassium chloride by a tissue homogenizer (Potter-Elvehjem, Thomas Scientific, Swedesboro, NJ, USA). The resultant homogenate was centrifuged for 10 min at 3000 rpm at 4 °C. Then, the supernatants were collected to assess testicular enzymes and antioxidant status. The last set of testis was fixed in 10% buffered formalin for histopathological and immunohistochemical evaluations of proliferating cell nuclear antigen (PCNA) and caspase-3 expression.

### 2.5. Semen Analysis

Directly after the rats were euthanatized, the cauda epididymis was removed and cut into small pieces using sterile scissors, then placed in 2 mL of 37 °C physiological saline. The resultant suspension was analyzed for motility, sperm concentration, and sperm abnormality. The epididymal suspension was examined under a microscope at 40× magnification by placing a drop on a clean glass slide that had been preheated to 37 °C and then covering the slide with another clean glass slide that had also been preheated to 37 °C. Many microscopical fields were examined to analyze approximately 200 sperms within 2–4 min of their extraction from the epididymis. The motile sperm cell percentage was assessed using a subjective scoring system ranging from 0 to 100 percent [52]. The sperm count was performed using a hemocytometer chamber slide after the sperm sample was diluted using normal physiological saline (1:4), then 4 drops of formalin (40%) were added to kill the spermatozoa [53]. The sperm cell concentration in 1 mL of semen sample was calculated using the following formula: *n* × 5 × 10 × dilution factor × 1000. Where *n* = the number of sperm in 0.1 mm^3^ of diluted semen [54]. The dilution factor used was 25 to prevent the overlapping of sperm cells and to facilitate sperm count for obtaining accurate results. The sperm abnormalities percentage was defined using eosin/nigrosine stained smears following the protocol of Filler [55].An amount of 10 µL of formalin-treated sperm solution was placed on a glass slide and accurately blended with 15 µL of 5% eosin solution and a drop of nigrosine. Then, the smears were made, dried in the air, and microscopically examined using higher magnification (400×). For each slide, one hundred spermatozoa were chosen randomly and analyzed for various anomalies in the head, neck/mid-piece, and tail.

### 2.6. Male Sex Hormones Measurements 

Male sex hormones were measured in serum samples by commercially available rat enzyme-linked immunosorbent assay (ELISA) kits, following the manufacturer’s directives. Cusabio Biotech Company supplied rat testosterone (Catalogue number: CSB-E05100r, sensitivity: <0.06 ng/mL. detection range: 0.13–25.6 ng/mL) and estradiol (Catalogue no.: CSB-E05110r, sensitivity: <40 ng/mL, detection range: 40–1000 pg/mL) assay kits (Wuhan, China). Kamiya Biomedical Company (Seattle, WA, USA) provided rat FSH (Catalogue number: KT-15332, sensitivity: 1.11 ng/mL, detection range: 2.47–200 ng/mL) and LH (Catalogue number: KT-21064, detection range: 0.37–30 ng/mL, sensitivity: 0.153 ng/mL) ELISA kits, which were used per the method of Zirkin and Chen [56].

### 2.7. Testicular Enzymes Evaluations

The levels of lactate dehydrogenase (LDH) in the testicular homogenates were measured using LDH Stanbio colorimetric kits (Stanbio Laboratories, TX, USA). LDH activity is directly proportional to the rate of nicotinamide adenine dinucleotide plus hydrogen (NADH) formation [57]. At 340 nm, the NADH optical density was measured. While sorbitol dehydrogenase (SDH) was estimated by rat-specific ELISA kits from MyBioSource (San Diego, CA, USA, Catalogue number: MBS166115, sensitivity: 0.62 ng/mL, detection range: 1.56–100 ng/mL) in line with the manufacturer’s directives.

### 2.8. Testicular Tissue Oxidative Stress Indices Assessment

The testicular content of reduced glutathione (GSH), catalase (CAT), and malondialdehyde (MDA) was evaluated by Bio-diagnostic colorimetric kits (Giza, Egypt, Catalogue number: GR 25 11, CA 25 17, and MD 25 29, respectively). CAT was estimated by the colorimetric technique through the reaction of CAT in the sample with a distinct amount of hydrogen peroxide (H_2_O_2_). An enzymatic reaction involving the conversion of 3,5-dichloro-2-hydroxybenzene sulfonic acid and 4-aminophenazone to a colored product is used to quantify the amount of H_2_O_2_ that remains. The amount of CAT in the sample is inversely proportional to the resulting chromogen color intensity [58]. The GSH content was determined by reducing 5,5’-dithiobis 2-nitrobenzoic acid (DTNB) with glutathione to generate a yellow compound whose absorbance at 405 nm can be used as an indicator of GSH concentration [59]. MDA levels were determined by their reaction with thiobarbituric acid in an acidic medium for 30 min at 95 °C, yielding a pink reactive product with an absorbance of 534 nm [60]. 

### 2.9. Determination of Testicular Zn Content

The Zn content of the testicular tissue of rats in different experimental groups was determined in line with the protocol of Choi et al. [61]. Initially, 1 g of testicular tissue was wet digested via mixing with 20 mL of HNO_3_ and HClO_4_ (4:1). Then, at 100 °C, the mixture was heated until the sample was completely dissolved. For the blanks, the same digestion steps were used. A Buck scientific model, 210 VGp flame with atomic absorption spectrophotometer, was used to determine absorbance and concentration. To control the technique’s accuracy and precision, the National Institute of Standards and Technology (NIST) standard reference material (NBS-bovine liver, No.1577 a) was used. The Zn recovery rate was 98%. Additionally, all glassware and plastic containers were washed with distilled water many times, submerged in 10% HNO_3_, and then rinsed in deionized water. Nitric acid, 65%, perchloric acid, and analytical-grade metals were used to generate a calibration curve. 

### 2.10. Histopathological Examination

Testes samples were extracted and fixed in neutral buffered formaldehyde for 48 h after rats were anaesthetized and euthanized. The fixed samples were processed by the standard paraffin embedding procedures. Based on the protocol of Suvarna et al. [62], 4 µm thick slices were hematoxylin and eosin (H and E)-stained. Then, a semi-quantitative scoring system for quantifying histopathological lesions was used following the protocol of Gibson-Corley et al. [63]. In brief, in randomly selected high power fields (HPF, 40 ×), an experienced non-biased pathologist blindly examined 50 tubular sections/testis per each animal in all experimental groups (10 animals/per group). The analyzed histopathological alterations were tubules with multinucleated giant cells, hyalinized tubules, and atrophied tubules. The detected lesions in seminiferous tubules were scored as follows: 0 = absence of lesion, 1 = 1–25% affected tubules; 2 = 26–50% affected tubules; 3 = 51–75% affected tubules; 4 = 76–100% affected tubules). The obtained scores were averaged and represented with a graph. Moreover, another score was made in line with the Johnsen’s scores criteria for analyzing the spermatogenesis alterations in the investigated groups [64], and the average Johnsen score/group was defined. 

### 2.11. Immunohistochemistry Evaluation 

The immunohistochemical technique was investigated using the method of Saleh et al. [65]. Goat polyclonal anti-PCNA (sc-9857, Santa Cruz Biotechnology, Dallas, TX, USA) and rabbit polyclonal anti-cleaved Caspase-3 (BioCare Medical, Cat. CP229C, Concord, CA, USA) were used with 1:2000 and 1:100 dilution, respectively. Briefly, after the deactivation of endogenous peroxidase enzyme through incubation with H_2_O_2_ (3% in absolute methanol) for 30 min at 4 °C, antigen retrieval for anti-PCNA only was used by Dako at 105 °C for 20 min. At room temperature for 60 min, 10% normal blocking serum (Sigma-Aldrich, Cat: A9647) was used to inhibit the nonspecific reaction. Then, the incubation of primary antibodies or normal goat IgG or normal rabbit IgG as negative controls was carried out overnight at 4 °C. For 60 min after a PBS wash, incubation with biotin-conjugated rabbit anti-goat IgG antiserum or goat anti-rabbit IgG antiserum (Histofine kit, Nichirei Corporation, Tsukiji, Tokyo, Japan) was performed based on the species’ primary antibody presented. Following a PBS wash, the sections were incubated with streptavidin-peroxidase conjugate (Histofine kit) for 30 min. Further, the streptavidin-biotin complex was incubated with a 3,30-diaminobenzidine tetrahydrochloride (DAB)-hydrogen peroxide (H_2_O_2_) solution at pH 7.0 for 3 min. Lastly, the sections were stained with Mayer’s hematoxylin counterstain. The sections were photographed using a microscope (Leica DM500) linked to a digital camera (Leica EC3, Leica, Germany). The Fiji image analyzer derived from image J software (National Institutes of Health, Bethesda, MD, USA) was used to quantitatively compute the area percentages of immunostaining reactions [66,67]. Ten randomly selected images from each group were deconvoluted into brown and blue colors of immuno-stain and hematoxylin, respectively. Then, the color threshold was adjusted and unified for all images to estimate area percentages of immunoreaction [68].

### 2.12. Real-Time Quantitative PCR (RT-qPCR) Analysis

Testis tissue total RNAs were extracted with Trizol Reagent (Thermo Fisher Scientific; Waltham, MA, USA) as per the manufacturer’s directions. For analysis of gene expression, two-step real-time PCR was conducted [69,70]. Briefly, cDNA was synthesized by a HiSenScript™ RH (-) cDNA Synthesis Kit (iNtRON Biotechnology Co., Seongnam, Republic of Korea) in a Veriti 96-well thermal cycler (Applied Biosystems, Foster City, CA, USA), followed by real-time PCR by an Mx3005P Real-Time PCR System (Conquer Scientific, Agilent Stratagene, Poway, CA, USA) through 5x HOT FIRE Pol EvaGreen qPCR Mix Plus (Solis BioDyne, Tartu, Estonia). All primers were synthesized by Sangon Biotech (Beijing, China), as shown in Table 1. Initial denaturation at 95 °C for 12 min was followed by denaturation at 95 °C for 40 cycles for 20 s, annealing for 30 s at 60 °C, and extension at 72 °C for 30 s. The relative expression levels of the aimed genes were normalized to GAPDH, and the relative folding changes in gene expression were assessed by the 2^−ΔΔCT^ method [71].

### 2.13. Data Analysis

The data normality was analyzed using the Shapiro–Wilk test, while Levene’s test was used to check the variance homogeneity. Then, the data were analyzed using one-way ANOVA, followed by the post hoc Tukey test, which determined significance when *p* < 0.05. Prism 7.0 GraphPad (Graph-Pad, San Diego, CA, USA) was used to conduct the statistical analyses.

## 3. Results

### 3.1. Changes in Body Weight Gain and Gonadosomatic Index in ACR and/or MO-ZNPs Administered Rats

Initially, rats that received only green synthesized MO-ZNPs displayed a significant (*p* = 0.02) increase in their body weight gain by 13.07% more than the control group (Table 2). In contrast, a significant (*p =* 0.02) reduction in body weight gain was recorded in the ACR group by 15.84% lower than in the control group. Instead, the ACR+MO-ZNPs group showed a significant (*p =* 0.02) increase in body weight gain compared with the ACR group. Moreover, no significant difference was recorded between the control and ACR+MO-ZNPs groups in their weight gain.

No significant change was recorded in the absolute testis weight in rats that received only MO-ZNPs compared with the control group. However, the absolute testis weight was significantly (*p* = 0.002) decreased in the ACR-exposed group by 35.53% lower than the control one. On the contrary, the ACR+MO-ZNPs group had a significantly (*p* = 0.002) higher absolute testis weight than the ACR group. Furthermore, no significant difference was found between the ACR+MO-ZNPs and control groups in their absolute testis weight. A non-significant difference was observed for the gonadosomatic index among all experimental groups (Table 2). 

### 3.2. Effect of ACR and/or MO-ZNPs Administration on Sperm Quality and Serum Levels of Circulating Reproductive Hormones

As revealed in Table 3, the individual administration of MO-ZNPs significantly (*p* < 0.001) increased the sperm count concentration by 22.15% compared to the control group. However, no significant changes were recorded in the sperm abnormalities and motility in the MO-ZNPs group compared to the control group. 

**Figure 1:**
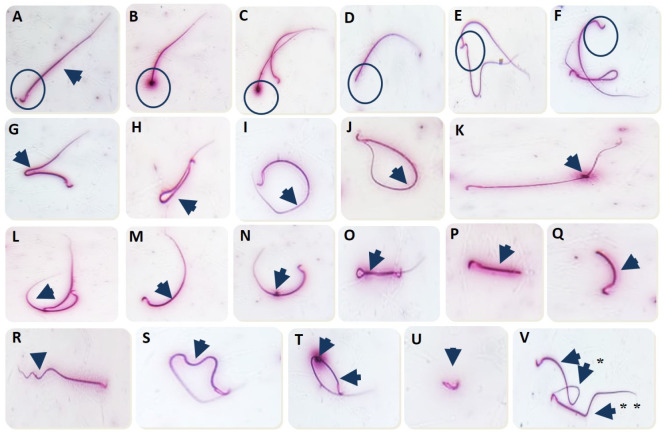
Abnormal spermatozoa induced in rats exposed to acrylamide. (**A**) Normal sperm; (**B**,**C**) Amorphous head; (**D**) Detached head; (**E**) Fused heads; (**F**) Bent head; (**G**,**H**) Bent tail; (**I**,**J**) Looped tail; (**K**) Protoplasmic droplet; (**L**,**M**) Curved tail; (**N**) Curved tail with protoplasmic droplet; (**O**) Coiled tail; (**P**) Short tail; (**Q**) Short curved tail; (**R**,**S**) Curled tail; (**T**) Looped tail with protoplasmic droplet; (**U**) Detached tail; (**V**) * Curved looped tail; (**V**) ** Broken tail.

Instead, the ACR-administered group revealed a significant (*p* < 0.001) decrease in sperm motility and sperm concentration by 70.59% and 74.85%, respectively, compared to the control group. Nonetheless, the sperm aberrations percentage significantly (*p* < 0.001) increased in the ACR-exposed rats by 188.08% compared to the control group (Table 3). As demonstrated in Figure 1, numerous abnormalities were apparent in the ACR-exposed rats, including tail (bent, looped, coiled, curved, short, curled, detached, broken, and curved with protoplasmic droplet) and head (amorphous, detached, fused, and bent). However, the MO-ZNPs+ACR-treated rats revealed a significantly (*p =* <0.001) elevated sperm motility and sperm count, while there was a significantly (*p* < 0.001) reduced sperm abnormalities percentage compared to the ACR-exposed group. 

The circulating levels of testosterone, estradiol, FSH, and LH were assayed to evaluate the impact of ACR on the pituitary–gonadal axis and the probable protective effect of MO-ZNPs (Table 3). The ACR-exposed rats showed a significant (*p* < 0.001) decrease in the serum testosterone and FSH by 92.66% and 49.32%, respectively, compared to the control rats. On the other hand, the serum estradiol (*p =* 0.004) and LH (*p =* 0.002) levels were significantly increased in the ACR-exposed group by 149.38% and 81.05%, respectively, compared to the control group. On the contrary, MO-ZNPs oral dosing significantly (*p* < 0.001) restored the ACR-induced reduction in testosterone compared to the ACR-exposed group, and the reduction percentage minimized to 21.56% lower than the control group. Moreover, the ACR-induced increment in estradiol (*p =* 0.004) and LH (*p =* 0.002) was significantly suppressed in the ACR+MO-ZNPs group compared to the ACR-exposed group. Furthermore, the improvement in estradiol and LH was marked to the extent that no significant differences were found compared to the control group. On the other hand, the FSH level tended to increase in the ACR+MO-ZNPs group but without significant change compared to the ACR-exposed group.

### 3.3. Effect of ACR and/or MO-ZNPs Administration on Testicular Biochemical Indicators and Zn Content

As displayed in Table 4, no significant change was noticed in the testicular enzyme levels when MO-ZNPs were administered alone compared to control rats. In contrast, the ACR-exposed group showed a significant (*p* < 0.001) increase in the LDH level by 67.19% compared to the control group, whereas co-treatment with MO-ZNPs significantly (*p* < 0.001) decreased the LDH level compared to the ACR group, and the increment was minimized to 20.42% higher than control values (Table 4). On the other hand, the SDH concentration was significantly (*p* < 0.001) decreased in the ACR group by 86.04% compared with the control group. Nevertheless, the ACR+MO-ZNPs co-administered rats displayed a significantly (*p* < 0.001) higher SDH than the ACR-exposed ones. 

As revealed in Table 4, rats that received MO-ZNPs only showed a significant (*p* < 0.001) increment in testicular GSH content by 59.48% compared with the control group. In contrast, the ACR-exposed group recorded a significant (*p* < 0.001) decrease in the testicular content of CAT and GSH by 94.66% and 47.07%, respectively, but a significant (*p* < 0.001) increase in MDA by 504.90% compared with the control group. In contrast, the CAT and GSH level in the ACR+ MO-ZNPs group significantly (*p* < 0.001) increased compared to the ACR-exposed group and became 65.11 % and 9.15 %, respectively, lower than the control group. Instead, the ACR-induced increment in testicular MDA content was significantly (*p* < 0.001) suppressed in the ACR+MO-ZNPs group compared to the ACR-exposed group and became 93.14% higher than the control group.

No significant change in the testicular Zn content was found between the MO-ZNPs, and ACR+ MO-ZNPs treated groups and the control group. On the contrary, the ACR-intoxicated rats had a significantly (*p* = 0.005) lower Zn content in their testes compared to MO-ZNPs, but did not significantly differ from control rats (Table 4).

### 3.4. Histopathological Assessment of Rat’s Testis

**Figure 2:**
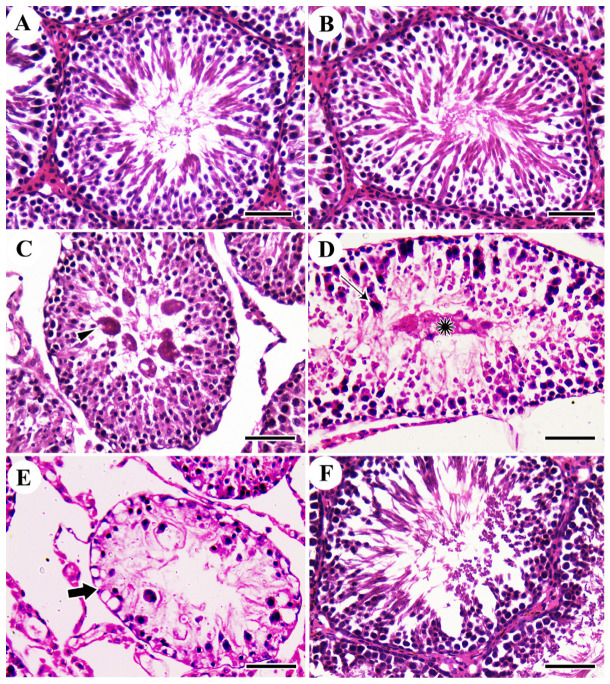
Histopathological examination of rat testis. (**A**) Control group; (**B**) *Moringa olifera* leaf extract (MO-ZNPs)-treated group; (**C**–**E**) Acrylamide (ACR)-exposed group showing multinucleated giant cell (arrowhead), necrosis of tubular epithelium (thin arrow), coagulative necrosis with luminal content hyalinization (aster), and atrophied seminiferous tubules with marked reduced numbers of necrotic germinal cells (thick arrow); (**F**) ACR+ MO-ZNPs. Scale bar = 50 µm.

**Figure 3:**
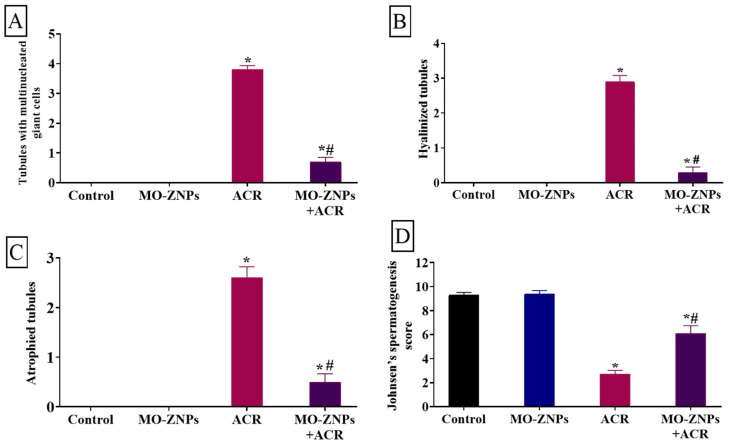
Changes in the testicular lesion score in rat testis of the different experimental groups (**A**–**C**): (**A**) Tubules with Multinucleated giant cells; (**B**) Hyalinized tubules; (**C**) Atrophied tubules; (**D**) Johnsen’s spermatogenesis score. Bars represent the mean ± SE. *n* = 10 replicates/treatment. * *p* < 0.05 vs. control. ^#^ *p* < 0.05 vs. ACR.

No histopathological testicular lesions were detected in the control and MO-ZNPs groups (Figure 2A,B). However, testicular samples isolated from the ACR group showed the formation of multinucleated giant cells in some seminiferous tubule’s lumen (Figure 2C). Moreover, coagulative necrosis of tubular epithelium with luminal content hyalinization was seen in some tubules (Figure 2D). In addition, some atrophied seminiferous tubules were detected, which were characterized by marked reduced numbers of necrotic germinal cells (Figure 2E). On the contrary, the ACR+MO-ZNPs-treated rats exposed a relative normal testicular architecture when compared with the control group (Figure 2F). Semi-quantitative analysis of the testicular histopathological lesions including multinucleated giant cells included tubules, hyalinized tubules, and atrophied tubules revealed that the ACR group had a markedly elevated level of testicular lesion score, more than the rats in the control and MO-ZNPs group. In comparison, the ACR+MO-ZNPs groups showed a significant decline in the testicular lesions score (Figure 3A–C). We analyzed the spermatogenesis alterations between various groups using Johnsen’s score methods. The testes of the ACR group revealed a marked reduction in Johnsen’s score compared with both the control and the MO-ZNPs groups. Moreover, a marked elevation in such score was detected in the testes of the ACR+ MO-ZNPs group compared with the ACR group. Furthermore, the testes of the ACR+ MO-ZNPs group displayed some degree of improvement in Johnsen’s score compared with that of the ACR group, and a significant reduction in the score compared with the control and MO-ZNPs group (Figure 3D).

### 3.5. Immunohistochemistry Assessment of Testis

**Figure 4:**
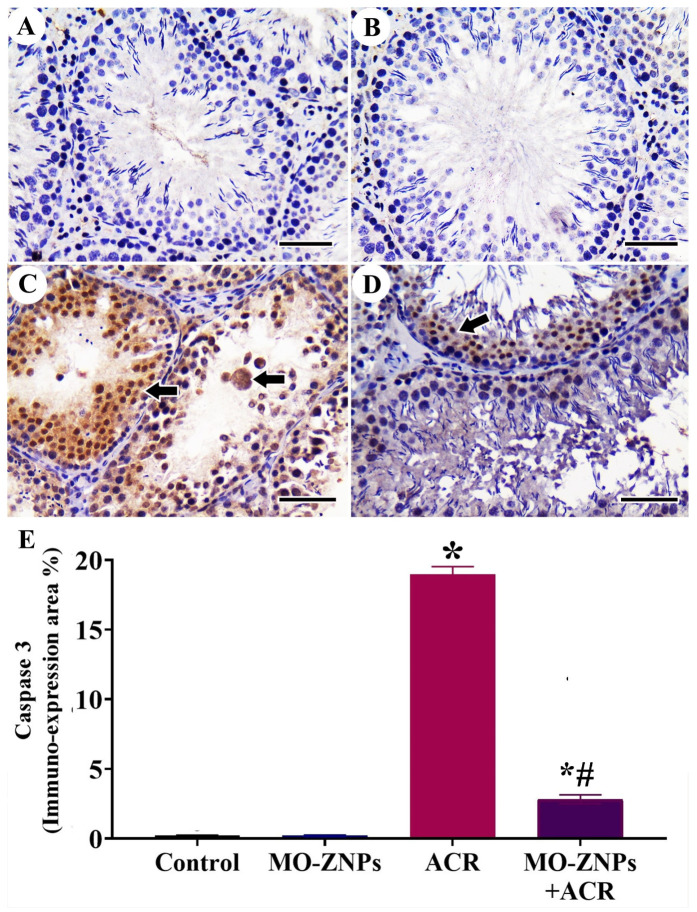
Immunohistochemical staining of rat testis by Caspase-3: (**A**) Control group; (**B**) ZnO group; (**C**) ACR group showing a high number of Caspase-3 reacted nuclei (arrows); (**D**) ACR+ MO-ZNPs group. Scale bar = 50 µm; (**E**) Changes in the immunohistochemical expression and quantitation of Caspase-3 in rat testis of the different experimental group. Bars represent the mean ± SE. *n* = 10 replicates/treatment. * *p* < 0.05 vs. control. ^#^ *p* < 0.05 vs. ACR.

The study of Caspase-3 expression in the testis of control and MO-ZNPs groups revealed mild expression (Figure 4A,B). On the other hand, samples extracted from ACR-treated rats showed the highest Caspase-3 expression in the spermatogenic nuclei (Figure 4C). Rats treated with ACR+MO-ZNPs showed decreased Caspase-3 expression in the spermatogenic nuclei (Figure 4D). The quantitative analysis for the area percentage of Caspase-3 immunohistochemical reacted nuclei illustrated a significantly elevated expression in ACR-treated rats compared to control and MO-ZNPs rats. This expression was markedly lowered in the ACR+ MO-ZNPs group (Figure 4E). 

**Figure 5:**
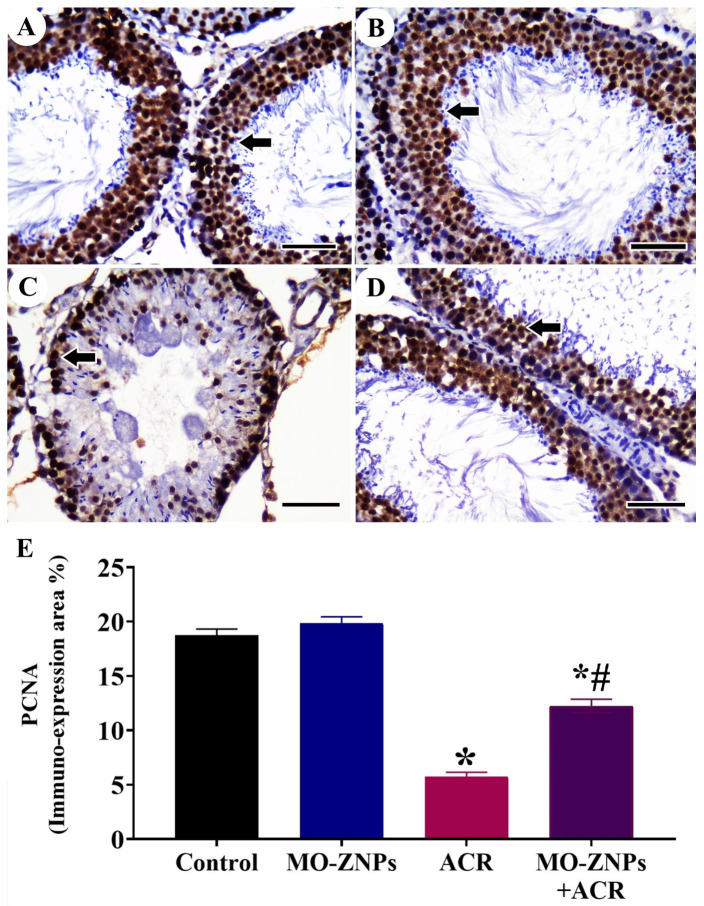
Immunohistochemical staining of rat testis by Proliferating Cell Nuclear Antigen (PCNA): (**A**) Control group and (**B**) MO-ZNPs group showing a high number of PCNA-reacted nuclei (arrows); (**C**) ACR group; (**D**) ACR+ MO-ZNPs group. Scale bar = 50 µm. (**E**). Changes in the immunohistochemical expression and quantitation of proliferating cell nuclear antigen (PCNA) in rat testis of the different experimental groups. Bars represent the mean ± SE. *n* = 10 replicates/treatment. * *p* < 0.05 vs. control. ^#^ *p* < 0.05 vs. ACR.

The exploration of testicular tissue for PCNA expression in the control and MO-ZNPs group displayed extensive expression in the nuclei of spermatogenic cells (Figure 5A,B). However, this extensive reaction was decreased in the ACR group (Figure 5C). The ACR+ MO-ZNPs group showed extensive restoration for PCNA expression in spermatogenic nuclei (Figure 5D). The nonparametric quantitative analysis for the area percentage of PCNA expression revealed a marked decreased PCNA expression in the ACR group compared to the control and MO-ZNPs groups. This reaction was markedly elevated in the ACR+ MO-ZNPs group (Figure 5E). 

### 3.6. Effect of ACR and/or MO-ZNPs Administration on Gene Expression Levels of Steroidogenesis-Related Genes

**Figure 6:**
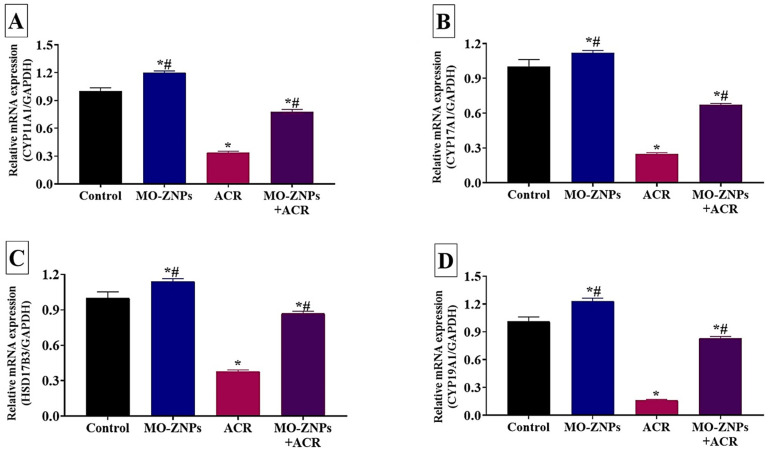
Effect of acrylamide (ACR) delivered orally (20 mg/kg) and/or zinc oxide nanoparticles synthesized by *M. oleifera* (MO-ZNPs) delivered orally (10 mg/kg bwt/day, 60 days) on mRNA expression of CYP11A1 (**A**), CYP17A1 (**B**), HSD17B3 (**C**), and CYP19A1(**D**). Bars represent the mean ± SE. *n* = 10 replicates/treatment. * *p* < 0.05 vs. control. ^#^ *p* < 0.05 vs. ACR.

**Figure 7:**
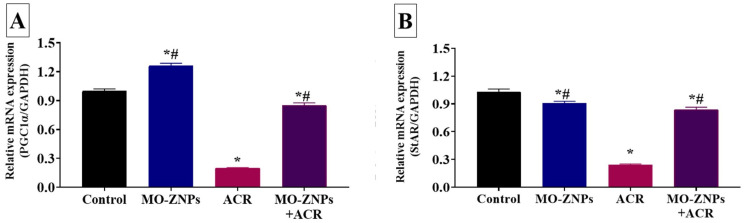
Effect of acrylamide (ACR) delivered orally (20 mg/kg) and/or zinc oxide nanoparticles synthesized by *M. oleifera* (MO-ZNPs) delivered orally (10 mg/kg bwt/day, 60 days) on mRNA expression of (**A**) peroxisome proliferator-activated receptor gamma coactivator 1-alpha (PGC-1α) and (**B**) steroidogenic acute regulatory protein (StAr) in the testicular tissues of male rats. Bars represent the mean ± SE. *n* = 10 replicates/treatment. * *p* < 0.05 vs. control. ^#^ *p* < 0.05 vs. ACR.

The changes in the mRNA expression levels of the steroidogenesis-related enzymes (StAR, HSD 17-B3, CYP11A1, CYP17A1, CYP19A1, and PGC1-α) in different experimental groups are shown in Figure 6 and Figure 7. The ACR-exposed group showed a significant (*p* < 0.001) reduction in gene expression levels of StAR, CYA11A1, CYA17A1, CYA19A1, 3β-HSD, and PGC-1α, relative to those in the control group. On the contrary, significantly higher expression levels of the steroidogenesis-related enzymes were recorded in the MO-ZNPs+ACR group compared to the ACR group.

## 4. Discussion

In the current study, ACR oral dosing for 60 days considerably impaired sperm quality and testicular function in rats, probably through meditating oxidative stress reactions and altering the steroidogenesis pathway. Nonetheless, the MO-ZNPs markedly counteracted the aforementioned ACR-associated injurious effects on male fertility. Body weight variation is a common parameter in toxicological studies of a substance’s effects on animal health [77]. Therefore, various toxicological studies have measured animals’ body weight and organ weight to determine how various toxins affect the body [78,79]. Herein, ACR-treated rats had an obviously lower body weight gain than the control group. Numerous animal models have demonstrated ACR’s ability to reduce body weight [80,81]. It has been revealed that daily exposure to ACR can cause a decrease in appetite drive or a slower rate of weight gain [82,83], which might be intermediated via augmented oxidative stress [84]. On the contrary, co-treatment with MO-ZNPs considerably counteracted the ACR-induced growth-lowering effect. This improvement could be attributed to the vital role of Zn in carbohydrate, lipid, and protein metabolism and energy utilization [85]. Moreover, Zn is necessary for the standard structural proteins, hormones, and enzyme functions needed for growth [86,87]. Furthermore, Imamoğlu et al. [88] proposed a role for Zn in synthesizing growth hormones.

Compared to control group rats, ACR exposure considerably reduced the absolute weights of testes. This decrease in testicular weight could be attributed to the ACR-induced histopathological lesions, including the atrophied seminiferous tubules and the reduced numbers of germinal cells. In contrast, the absolute testicular weight was significantly improved in the ACR and MO-ZNPs co-administered group. The recorded improvement in the histological architecture of MO-ZNPs+ACR rat testis could be responsible for regaining their weight.

In the current study, the ACR exposure (20 mg/kg for 60 days) substantially disturbed sperm parameters, as reflected in a diminution in epididymal sperm quality and count, reduced sperm motility, and increased sperm abnormalities, chiefly head and tail malformations. The spermatogenesis impairment has been known as a main cause of reduced semen quality [89]. Herein, the microscopical findings in ACR-exposed rat testicular tissues revealed necrosis of the tubular epithelium of seminiferous tubules with luminal content hyalinization. In addition, some atrophied seminiferous tubules were discovered, distinguished by a noteworthy reduction in necrotic germinal cells. Similarly, ACR exposure impaired spermatogenesis in earlier experimental studies [12,19]. These impairments could be caused by ACR-prompted epithelial cell disintegration in the seminiferous tubules and ACR interference with the kinesin motor protein in the sperm flagella [90]. In contrast, MO-ZNPs significantly repaired the ACR-induced impairments in sperm characteristics. Comparable enhancement of sperm parameters has been associated with ZNPs [91]. The recorded rise in the activity of SDH enzyme in the MO-ZNPs+ACR group could partly share in the improved sperm motility because of its known role in affording the energy required for sperm [92]. At the same time, Zn is a vital component of SDH [93]. In addition, the Zn’s antioxidant activity could also play a role in sperm membrane stabilization and consequently increase its viability and motility and reduce the abnormalities [94]. In this regard, Zn has been reported to prevent iron from entering the ROS generation cycle or Fenton reactions, thereby protecting the membrane’s lipid structure from free radical attack [94]. In this regard, the co-administration of MO-ZNPs+ACR restored the testicular tissue architecture. Similarly, Erfani Majd et al. [91] found that administering different forms of ZNPs, particularly the green synthesized form, significantly repaired the histological tissue alterations that resulted from cisplatin administration. Furthermore, Zn aids in the transcription of many factors involved in spermatogenesis [95].

Herein, exposure to ACR for 60 days notably decreased the serum levels of FSH and testosterone while increasing estradiol and LH. Comparable ACR-induced disturbance in male reproduction hormones was reported in the studies of Shahrzad et al. [11] and Yildrim et al. [96]. The ACR-induced decrease in testosterone could be due to Leydig cell weakness caused by excessive LH secretion due to ACR exposure [90]. The inverse relationship between testosterone and estradiol could be due to testosterone-to-estradiol conversion via increased aromatase expression in adipose tissue [75]. In vitro studies suggested that even small levels of ACR decrease the activity of steroidogenic enzymes such as HSD17B3, reducing testosterone production [97]. In the current study, StAR, HSD17B1, CYP11A1, CYP17A1, and CYP19A1 mRNA expressions were lower in the ACR group than in the control group, which is similar to the previous findings of Yildrim et al. [96]. This could imply that ACR inhibits testosterone synthesis via altering STAR, CYP11A1, CYP17A1, CYP19A1, 17β-HSD, and PGC1a mRNA expressions. The decreased activity levels of testicular 17β-HSDs and StAR in ACR-exposed rats may indicate improper cholesterol channeling and decreased steroidogenesis [98]. On the other hand, MO-ZNPs considerably corrected the ACR-induced disturbances in male reproductive hormone levels. The earlier findings reflected Zn’s repro-protective nature [46] and its significance in the normal function of the hypothalamus–pituitary–gonadal axis [99,100]. Furthermore, MO-ZNPs significantly counteracted the ACR-induced decrease in STAR, CYP17A1, 17β-HSD, and PGC1a mRNA expressions in testicular tissues, thereby suppressing the ACR-induced reduction of testosterone synthesis. Similarly, Erfani Majd et al. [91] demonstrated that green synthesized ZNPs upregulated STAR and CYP11A1 gene expressions of cisplatin-treated rats. Furthermore, a significant increase in StAR expression was recorded in mouse Leydig cells exposed to ZNPs [101]. The steroidogenesis effect of ZNPs may be related to the ease with which ZNPs pass through the blood–brain barrier and blood–testis barrier, as well as its effect on LH level [102]. In addition, Zn has been reported to increase the pituitary gland’s release of LH and FSH, stimulating testosterone production. Zn also constrains the aromatase enzyme, which converts testosterone into extra estrogen [103]. Furthermore, Zn plays a vital role in the pathways that control steroid synthesis [104].

The current experiment showed a substantial increase in testicular LDH but reduced SDH levels in ACR-exposed rats. The increased LDH enzyme could be due to membrane integrity disruption caused by the negative effects of high ROS levels due to ACR [105]. Meanwhile, the reduction in SDH observed in this study suggests that ACR exposure may cause an energy imbalance in the sperm cell, which explains the impaired sperm motility and viability. However, MO-ZNPs co-administration normalized the levels of testicular enzymes, indicating its potent protective role for biological structures against free radicals. MO-ZNPs could guard cell membrane integrity against ROS via their antioxidant activity and reduce LDH leakage [106]. Furthermore, Zn acts as a cofactor in the activities of SDH [107].

In the present study, ACR dosing significantly diminished the testicular CAT and GSH activity levels but increased MDA content. The involvement of oxidative stress in ACR-induced testicular toxicity has been reported earlier [19]. The ACR-induced oxidative stress is mainly linked to the covalent adducts formation of it or its epoxide metabolite, glycidamide, with the cellular macromolecules or their interaction with the thiol groups found in most biomolecules [108]. Furthermore, ACR has been reported to react with GSH directly, form glutathione S-conjugates, and initiate intracellular electrophile metabolism [109]. Nevertheless, MO-ZNPs significantly restored the depleted CAT and GSH and reduced the increment in MDA level resulting from ACR exposure. Similarly, MO-ZNPs combated the oxidative stress from rotenone [46] and cisplatin [91]. Akintunde et al. [46] suggested that green ZNPs could act as a cofactor of SOD to stimulate metallothionein production, a potent hydroxyl radical scavenger. Furthermore, Celino et al. [110] verified the efficacy of Zn in protecting the sulfhydryl group against oxidation and maintaining intracellular GSH levels.

In the current study, no significant difference was found in total Zn contents between the MO-ZNPs and the control group. This could be because the sample was collected 24 hrs. after the final gavage. Likewise, Dahran et al. [16] reported that a non-significant increase in brain Zn content was apparent after repeated ZNPs exposure. However, MO-ZNPs supplementation in the ACR+MO-ZNP-treated rats increased testicular Zn levels compared to the ACR group. These findings suggested that Zn dosing could ameliorate the ACR-induced testicular injury by re-establishing Zn levels in testicular tissue. Similarly, several earlier studies confirmed that Zn supplementation is the basis for alleviating chemotherapy-induced testicular damage by restoring the testicular Zn content and enhancing the testicular functions [111,112].

The current immunohistochemical findings revealed that Caspase-3 expression was significantly higher in the ACR-exposed group’s testes than in the control group. Comparable ACR-associated apoptotic activity was earlier recorded in the studies of Adelakun et al. [113] and Farag et al. [19]. The ACR-induced oxidative stress has been linked to the activation of the mitochondrial apoptotic pathway [114]. In contrast, MO-ZNP co-administration in ACR-exposed rats decreased testicular Caspase-3 expression, which could be ascribed to increased antioxidant activity. In addition, the antiapoptotic effect of ZNPs was previously reported by Anan et al. [106] and Tian et al. [115]. In this regard, ZNPs have been reported to decrease apoptosis-inducing factors and cytochrome c, which are internal signals for apoptosis induction in the cells [116]. On the other hand, Chung et al. [117] investigated the cytotoxic potential of ZNPs synthesized from *Eclipta prostrata* on HepG2 cells and discovered that 100 mg/mL resulted in substantial cytotoxic effects, while activating Caspase-3 established the cells’ apoptotic features. Nonetheless, ZNPs could exert their beneficial pharmacological effects without exerting cytotoxic impacts on the host cells [118], particularly at low concentrations [119].

PCNA is a nuclear protein that binds to DNA polymerase to promote DNA replication [120]. Hence, it is a reliable tool for identifying the loss of germinal cells, which is connected to decreased PCNA and compromised DNA synthesis [121]. Herein, ACR reduced germ cell multiplying in rat testes, as evidenced by decreased PCNA immune expression. ACR’s reduced expression of PCNA has recently been documented [113]. ACR can generate ROS, which contributes to DNA damage. ACR-accompanying apoptosis, along with diminished cell multiplying, could be the cause of testicular dysfunction and decreased sperm viability and mobility [122]. On the other hand, the recorded PCNA expression increase in MO-ZNPs-treated groups could be owed to DNA repair activities, increased cell cycle progress, and decreased apoptosis. Furthermore, the MO-ZNPs antioxidant activity may prevent apoptotic cell death and promote cell proliferation, and treatment with MO-ZNPs may compensate for ACR-induced loss of testicular germ cells.

Despite being the first study to show the beneficial role of green synthesized ZNPs using *M. olifera* extract in reducing ACR-associated testicular injury and altered spermatogenesis. However, the probable underlying mechanisms need further investigation, particularly the role of other genes in steroidogenesis and other apoptotic pathways. Moreover, the probable modulatory role of MO-ZNPs in reducing ACR-induced autophagy pathway alteration needs to be evaluated.

## 5. Conclusions

The present study’s results verified that the green synthesized MO-ZNPs by *M. olifera* extract could be a protecting candidate for ACR-associated adverse impacts on the fertility of male rats. These repro-protective effects could be mediated by improving testicular antioxidant activity, reducing testicular lipid peroxidative damage, balancing male sex hormones, maintaining testicular Zn content, and controlling steroidogenesis and apoptotic pathways. Consequently, further studies on humans are highly needed to determine if MO-ZNPs could be used as a protective strategy in people who are likely to be exposed to ACR, such as those who work in the manufacturing industry. Additional research on the other potential mechanisms underlying MO-ZNPs protection is also required.

## Figures and Tables

**Table 1:** Primer sequences, accession number, and product size for the quantitative RT-PCR for the analyzed genes in the testicular tissue.

Genes	Primer Sequences	Product Size /bp	GenBank Accession Numbers	References
StAR	5’-CCCAAATGTCAAGGAAATCA-3’	187	NM_031558.3	[72]
	3’-AGGCATCTCCCCAAAGTG-5’			
CYP11A1	5’-AAGTATCCGTGATGTGGG-3’	127	NM_017286.3	[72]
	3’-TCATACAGTGTCGCCTTTTCT-5’			
CYP17A1	5’-TGGCTTTCCTGGTGCACAATC-3’	90	NM_012753.2	[72]
	3’-TGAAAGTTGGTGTTCGGCTGAAG-5’			
CYP19A1	5’-GCTGAGAGACGTGGAGACCTG-3’	178	NM_017085.2	[73]
	3’-CTCTGTCACCAACAACAGTGTGG-5’			
HSD17B3	5’-AGTGTGTGAGGTTCTCCCGGTACCT-3’	161	NM_054007.1	[74]
	3’-TACAACATTGAGTCCATGTCTGGCCAG-5’			
PGC1-α	5’-ATGTGTCGCCTTCTTGCTCT-3’	180	NM_031347.1	[75]
	3’-ATCTACTGCCTGGGGACCTT-5’			
GAPDH	5’-GGCACAGTCAAGGCTGAGAATG-3’	143	NM_017008.4	[76]
	3’-ATGGTGGTGAAGACGCCAGTA-5’			

StAr: steroidogenic acute regulatory protein; CYP11A1: cytochrome P450 Family 11 Subfamily A; CYP17A1: cytochrome P450 Family 17 Subfamily A; CYP19A1: cytochrome P450 Family 19 Subfamily A; HSD17B3: 17-beta hydroxysteroid dehydrogenase 3 Member 1; PGC-1α: peroxisome proliferator-activated receptor gamma coactivator 1-alpha; GAPDH: glyceraldehyde-3-phosphate dehydrogenase.

**Table 2:** Effect of green synthesized zinc oxide nanoparticles produced by *Moringa oleifera* leaf extract (MO-ZNPs) on body weight change and testes weights in acrylamide (ACR)-exposed rats.

Experimental Groups	Body Weight Gain (g)	Absolute Weight of Testis (g)	Gonadosomatic Index
Control	168.33 ± 14.75	1.97 ± 0.08	1.19 ± 0.12
MO-ZNPs	190.33 * ± 2.60	2.30 ± 0.06	1.21 ± 0.01
ACR	141.67 * ± 7.54	1.27 * ± 0.09	0.89 ± 0.04
ACR+MO-ZNPs	164.33 ^#^ ± 1.45	1.83 ^#^ ± 0.20	1.12 ± 0.13

Values are represented as the mean ± SE. *n* = 10 replicates/treatment. * *p* < 0.05 vs. control. ^#^ *p* < 0.05 vs. ACR.

**Table 3:** Effect of green synthesized zinc oxide nanoparticles produced by *Moringa Oleifera* leaf extract (MO-ZNPs) on sperm parameters and serum hormone levels of acrylamide (ACR)-exposed rats.

Estimated Parameters	Experimental Groups			
	Control	MO-ZNPs	ACR	ACR+MO-ZNPs
**Sperm parameters**				
Sperm motility (%)	85.00 ± 2.89	94.00± 1.00	25.00 * ± 2.89	65.00 *^#^ ±2.89
Sperm count (sp.cc/mL × 125 × 10^4^)	55.67 ± 2.96	68.00 ^*^ ±1.73	14.00 * ± 2.08	39.33 *^#^ ±3.48
Sperm abnormalities (%)	16.78 ± 1.24	12.66± 0.67	48.34 * ± 3.84	27.78 *^#^ ±0.87
**Serum hormonal analysis**				
Testosterone (ng/mL)	2.18 ± 0.06	2.33 ± 0.04	0.16 * ± 0.03	1.71 *^#^ ± 0.12
FSH (ng/mL)	5.13 ± 0.31	5.92 ± 0.35	2.60 * ± 0.12	3.10 * ± 0.20
Estradiol (pg/mL)	42.03 ± 0.09	42.33 ± 1.20	60.00 * ± 4.00	46.83 *^#^ ± 3.03
LH (ng/mL)	1.90 ± 0.16	1.61 ± 0.03	3.44 * ± 0.35	2.19 *^#^ ± 0.24

FSH: Follicle-stimulating hormone; LH: luteinizing hormone. Values are represented as the mean ± SE. *n* = 10 replicates/treatment. * *p* < 0.05 vs. control. ^#^ *p* < 0.05 vs. ACR.

**Table 4:** Effect of green synthesized zinc oxide nanoparticles produced by *Moringa oleifera* leaf extract (MO-ZNPs) on testicular enzymes, antioxidant status, and Zn content in the testis of acrylamide (ACR)-exposed rats.

Estimated Parameters	Experimental Groups			
	Control	MO-ZNPs	ACR	ACR+MO-ZNPs
**Testicular enzymes**				
LDH (U/L)	125.18 ± 1.50	94.43 ± 5.44	209.29 * ± 10.61	150.74 ^#^ ± 7.05
SDH (ng/mg)	42.12 ± 2.25	43.47 ± 1.95	5.88 * ± 1.52	30.47 *^#^ ± 0.93
**Antioxidant parameters**				
CAT (ng/mg)	10.29 ± 0.73	11.07 ± 0.08	0.55 * ± 0.04	3.59 *^#^ ± 0.47
GSH (ng/mg)	130.13 ± 4.33	207.53 * ±12.84	68.88 * ± 4.71	142.04 ^#^ ± 4.75
MDA (nmol/mg)	1.02 ± 0.04	0.49 ± 0.04	6.17 * ± 0.72	1.97 ^#^ ± 0.16
**Zn residues (ppm)**	11.90 ± 0.44	13.09 ^#^ ± 0.52	10.52 ± 0.06	11.33 ± 0.15

LDH: lactate dehydrogenase; SDH: Sorbitol dehydrogenase; CAT: Catalase; GSH: Reduced glutathione; MDA: Malondialdehyde. Values are represented as the mean ± SE. *n* = 10 replicates/treatment. * *p* < 0.05 vs. control. ^#^ *p* < 0.05 vs. ACR.

## Data Availability

All datasets generated for this study are included in the article.
